# Influencing Mechanisms of Urban Heat Island on Respiratory Diseases

**Published:** 2019-09

**Authors:** Huanchun HUANG, Hailin YANG, Xin DENG, Peng ZENG, Yong LI, Luning ZHANG, Lei ZHU

**Affiliations:** 1. Department of Urban Planning, School of Landscape Architecture, Nanjing Forestry University, Nanjing, China; 2. Department of Urban Planning, School of Architecture, Tianjin University, Tianjin, China; 3. People’s Hospital of Jiangsu Province, Nanjing Medical University, Nanjing, China; 4. Department of University Foreign Language Teaching Section, School of foreign Studies, Nanjing Forestry University, Nanjing, China; 5. Center for Educational Research, Seoul National University, Seoul, South Korea

**Keywords:** Urban heat island intensity, Respiratory disease, Landscape pattern, Evolutionary characteristic

## Abstract

**Background:**

Urban heat island (UHI) is being intensified with the progress of urbanization. Meanwhile, respiratory diseases caused by high temperature become common. This study explores the influences of UHI on respiratory diseases (J00-J99) and the evolutionary characteristics of the spatial pattern of such influences.

**Methods:**

The pattern–process–function and the influencing mechanism of UHI on respiratory diseases were evaluated through landscape pattern indexes from 1992 to 2018 in Tianjin, China. The basis was on data from Landsat TM/OLI/TIRS remote-sensing images, meteorological stations, and mortality of respiratory diseases.

**Results:**

The fluctuating influence of UHI on the respiratory diseases in Tianjin has increased from 1992 to 2018, showing a significant phase-based characteristic. During 2011–2018, the influence has soared greatly, and mortality risk has increased by 101%, and the influenced area has reached 349 km^2^. Furthermore, the regional space clustered, and the influenced patches are in irregular shape, and the highly influenced area is enlarged. Moreover, the indexes of the landscape level of the influenced areas all decrease. The patches at all levels are fragmented and distributed discontinuously. Spatially, the influenced areas gradually extend from the urban center to the suburbs.

**Conclusion:**

UHI causes a higher mortality of respiratory diseases because it increases daily average air temperature in summer. With respect to landscape pattern, the influenced areas at low level is highly interconnected and relatively concentrated, whereas the influenced area at high level is distributed in clusters. In general, the influenced area is fragmented and discontinuously distributed in urban center.

## Introduction

Urban heat island (UHI) becomes serious with the quick progress of urbanization. Therefore, temperature in urban areas in summer keeps increasing. In the past century, the average surface temperature has increased by 0.5 °C–0.8 °C. According to the estimation by the National Meteorological Bureau (NMB) of China, the average temperature would increase by 2.2 °C–3.3 °C by 2050 (1). Global warming is accelerating and bringing a series of disasters (2–3). In addition to death, high temperature and heat waves induce various diseases in respiratory, urinary, circulatory, and nervous systems. Respiratory diseases are the most severe one (4–5). Many factors contribute to the respiratory diseases. Studies in recent years focused on the effects of air temperature (6). However, existing studies only focus on epidemiological investigation, and cannot explain the space–time interaction mechanism between air temperature and respiratory diseases.

Scholars have conducted numerous studies on the relationship of temperature and respiratory diseases. The admission rate of patients (>75 years old) with respiratory diseases increased by 3% with every 1 °C above the threshold temperature (the maximum daily average temperature = 29.5 °C) (7). The daily average air temperature is in a V-shaped correlation with the emergency visits for respiratory diseases. When the daily average air temperature is lower/higher than the optimal temperature, the excess risk of emergency visits for respiratory diseases is 3.75%/1.54% of the total population with every 1 °C above/below the daily average air temperature, respectively (8). However, high temperature and heat waves have the most serious impact on human health. The incidence of upper respiratory infection at the Summer Solstice reaches 56.3 people/d (9).

The spatial–temporal distribution of urban landscape pattern forms up UHI, which exerts an influence driven by multi-dimensions like quantity and quality on the UHI (10). The number of patches (NP) and patch density (PD) decreases at the class level, whereas the percent of landscape (PLAND) continuously increase with the progress of urbanization. At the landscape level, aggregation index (AI) and contagion index (CONTAG) generally increase, whereas Shannon diversity index (SHDI) and Shannon’s evenness index (SHEI) decrease (11). There is an evident correlation between the incidence of Lyme disease and landscape fragmentation and temperature by studying landscape fragmentation and meteorological factors (12), and CONTAG influences the spread of West Nile virus (WNV) (13). Thus, the spatial–temporal distribution of UHI causes certain influences on diseases. However, the relationship of UHI landscape pattern and respiratory diseases remains unclear.

Therefore, the purpose of the study was to establish the grading standards for the influences of UHI on respiratory diseases and assess the effects of UHI on the spatial distribution of respiratory diseases by landscape pattern indexes.

## Materials and Methods

### Data Resources and Preprocessing

#### Data Resources

The original data of LST were collected from the Landsat images of urban areas in Tianjin in 1992, 1999, 2001, 2006, 2009, 2011, 2013, and 2018. Images were collected during 10:30–11:00 in July and August in each sampling year. The average wind speed two days before the image collection was smaller than 2.3 m/s, and no precipitation occurred on the day of image collection. At the imaging moment, the area in the study was with good atmospheric visibility, imaging conditions and cloudless. Remote sensing image data were sufficiently high-quality for temperature retrieval.

Meteorological data were collected from 18 stations in urban area and 6 stations in suburbs of Tianjin and 350 stations in the neighboring Beijing, China on the same latitude. All meteorological stations selected observation sites with small interference from surrounding buildings, vegetation, hardening ground, and artificial facilities on UHI, to assure data accuracy.

Data on mortality of respiratory diseases in Tianjin, which include sex, date of birth, date of death, age, and primary cause of death, were obtained from the analysis of the aforementioned variables by Disease Control and Prevention Center of China (14). Respiratory diseases (J00-J99) were selected in accordance with the *International Classification of Diseases*, 10th edition (ICD-10).

#### Data preprocessing

The remote sensing images were registered, and their geometric accuracy was calibrated. Satellite images were calibrated to 0.5 m resolution using quadratic polynomials. Gray interpolation was performed by cubic convolutions. Error was controlled within a pixel. Later, data were resampled to 30 m resolution. The data were projected to WGS_1984_UTM_50N coordination system to maintain the consistency. Finally, a database was constructed by ArcGIS software. The remote sensing image data in different periods were extracted and analyzed.

### Research Methods

#### Land surface temperature retrieval

In this study, the atmospheric calibration method was applied. First, this method estimates the influence of atmosphere on surface thermal radiation. Second, such influence is subtracted from the total thermal radiation received by the satellite sensor (15), thereby the surface brightness temperature is obtained. Third, the surface brightness temperature is converted into its corresponding LST. The formula is expressed as follows:

$$T_{\text{s}}=K_{2}/\ln\left(K_{1}/B\left(T_{\text{s}}\right)+1\right)$$

where (*T_s_*) is the surface brightness temperature (*K*); (*K*_1_ and *K*_2_) are calibration constants of the sensor, and their numerical values can be acquired from the metadata of images: (*K*_1_ =774.89*W*/(m^2^·*μm* · *sr*) and (*K*_2_ =1231.08*K*); (*B(T_s_*) is the radiation brightness of the black body with the temperature of (*T_s_*) in the thermal infrared band.

The formula is expressed as follows:

$$B\left(T_{\text{s}}\right)=\left[L_{\lambda }-L_{↑}-\tau \left(1-ɛ\right)L_{↓}\right]/\tau ɛ$$

where (*L_λ_*) refers to the thermal infrared radiation brightness value received by the satellite sensor and (*ε*) is the land surface emissivity (LSE), (*τ*) the transmittance of atmosphere in thermal infrared band, (*L*_↓_) the downward radiation brightness of atmosphere, (*L*_↑_) the upward radiation brightness of atmosphere and (*τ*, *L_↓_*, and *L_↑_*) are acquired from the official website of NASA (*τ* = 0.9, *L_↓_* = 1.37*W*/(*m*^2^· *μm* · *sr*), and *L_↑_* = 0.78*W*/(*m*^2^· *μm* · *sr*), respectively). When solving (*ε*), surface can be divided into three average types: water, urban area, and natural surface (16). The formulas of (*ε*) in each type are presented as follows:

$$\begin{array}{l} {ɛ}_{\text{w}}=0.995\\ {ɛ}_{\text{b}}=0.9589+0.086P_{\text{v}}-0.0671P_{v}^{2}\\ {ɛ}_{\text{s}}=0.9625+0.0641P_{\text{v}}-0.0461P_{v}^{2} \end{array}$$

where *P_v_* is the vegetation cover and can be calculated as:

$$P_{\text{v}}=\left(N-N_{\text{s}}\right)/\left(N_{v}-N_{s}\right)$$

where (*N*) is the normalized vegetation index of the whole study area. (*N*_s_) is the NDVI value of pixels of complete bare soil or zero-vegetation coverage and (*N*_v_) the NDVI value of pixel completely covered by vegetation (17). (*N_s_* and *N*_v_) apply the empirical values of 0.05 and 0.07, respectively (18).

### Grading of influences of UHI on respiratory diseases mortality

Studies prove that high-temperature and high-humidity environments inhibit droplet transmission but also provide appropriate conditions for survival and spread of relevant viruses and bacteria, such as parainfluenza virus 3. Such viruses and bacteria deposit in the respiratory tract, inducing respiratory diseases (19–20). The maximum temperature (approximately 32 °C) was reached in July and August. Long-term exposure to such environment can cause temporary obstacles to thermoregulatory mechanism, which is a potential cause for respiratory diseases (21). According to previous studies, the daily average temperature threshold of influences on the respiratory disease mortality in Jinan of China, in the same climatic zone of Tianjin, is 31 °C. The respiratory disease mortality increases by 25.3% with every 1 °C rise of air temperature (14).

In this study, the daily average temperature at suburb observation points in Tianjin in July and August during 2008–2018 was 27.5 °C according to the meteorological data and responses of human body to high temperature. The difference between UHI intensity and daily average temperature (3.5 °C) was applied as the reference value of UHI intensity. The influences of UHI on the mortality of respiratory diseases were divided into five levels (Table 1).

**Table 1:** Grading of influences of UHI on respiratory diseases mortality

***Grading of influenced areas***	***UHI intensity /°C***	***Growth of mortality /%***	***Physiological reaction of human body***
Level-0	3.5	0	The human body feels slightly uncomfortable
Level-1	3.5–4.5	0–25.3	The human body feels uncomfortable, with the physiological manifestation of dry cough
Level-2	4.5–5.5	25.3–50.6	The human body feels extremely uncomfortable, with the main physiological manifestation of cough and expectoration
Level-3	5.5–6.5	50.6–75.9	Reaching the highest temperature threshold of death of respiratory diseases, the incidence of respiratory diseases increases accordingly
Level-4	6.5–7.5	75.9–101.2	People may suffer from dyspnea. The mortality of respiratory diseases increases

**Notes:** The calculation of UHI intensity was done with the daily average temperature in 32 suburb rural sites as reference

### Evaluation method by landscape pattern indexes

Landscape pattern refers to the arrangement and combination of landscape elements in different sizes and shapes in a space, such as class, number, and spatial distribution and configuration of landscape elements (22). Heterogeneity, diversity, and dispersion degrees are described by the comprehensive index method to provide key reference for the reasonable planning of the landscape. Landscape pattern indexes include patch, class, and landscape levels. PLAND, PD, and cohesion (COHESION) of the class level, as well as AI, CONTAG, SHDI, and SHEI of the landscape level are selected according to the characteristics of the study area.

### Survey of Research Areas

Tianjin (38°33′–40°15′, 116°42′–118°03′E), is next to the Bohai Sea on the east and Yanshan Mountain on the north, adjacent to Beijing and located in the North Temperate Zone. The annual average temperature is approximately 14°C, with the highest in July. Most regions in Tianjin are plain, accompanied with a few mountains and hills.

**Fig. 1:**
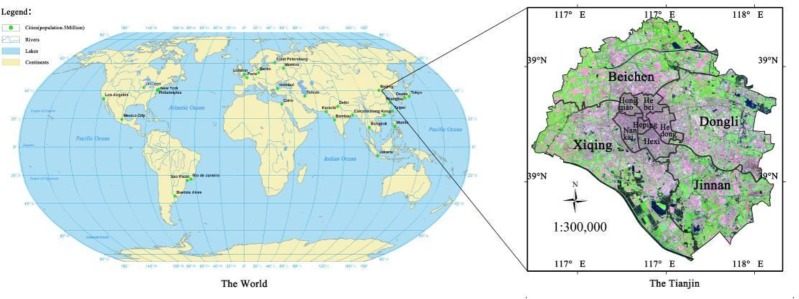
Study area

As a city with the most economic competitiveness in the world, Tianjin is a second-tier city in the world and is among the top 50 in Asia. It has maintained high-efficiency urbanization in the past three decades. Urban development in Tianjin is typical across the entire globe. Six central urban areas and four suburb regions with the most rapid progress of urbanization in Tianjin are selected as the study area, which covers a total area of 2,080 km^2^ (Fig. 1).

## Results

### Spatial Characteristics of the Influences of UHI Intensity on Respiratory Diseases

**Fig. 2:**
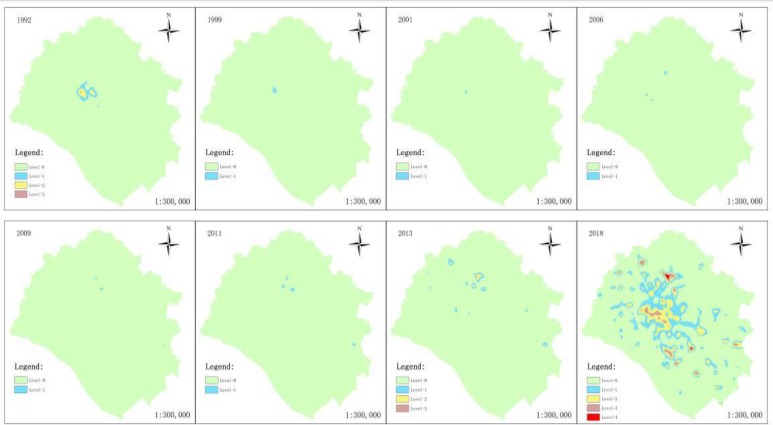
Zoning of influenced areas during 1992–2018

Fig. 2 shows the influence levels of UHI intensity on respiratory diseases from 1992–2018. The influence increased from level-3 to level-4. The mortality of respiratory diseases increased by up to 101%, and the region with increased respiratory diseases mortality covered 349 km².

The influenced area mainly ranged from level-1 to level-3 in 1992. The area of level-2 was the largest and mainly in the central region, because of heat concentration and poor ventilation caused by high-density low-rise buildings in the central regions. During 1999–2011, only small level-1 regions were present, and the number of patches continuously increased. Most patches were in peripheral regions of the central area. The number of influenced patches in 2013 was equal to that in 1992, but the center of influenced area shifted to peripheral industrial parks. In 2018, level-4 influenced area emerged as the mortality of respiratory diseases dramatically increased. The patches in all affected areas spread dramatically to central region and suburbs. Overall, the seriousness of impact on the influenced areas continuously increases and the influenced areas expand with the progress of urbanization. UHI has lasting serious influence on respiratory diseases.

### Landscape Pattern Analysis on Influences of UHI on Respiratory Diseases

#### Changes in class level of landscape pattern

Table 2 shows the changes in PLAND in 1992–2018. Level-0 accounted for the highest proportion, but its decrease was fluctuating. On the contrary, proportions of other levels continuously increased. Therefore, the influences of UHI intensity on respiratory diseases were intensified, and the influenced areas kept expanding. Particularly, Level-1 and Level-2 expanded most drastically. In detail, they expanded by 15.8 times and 14.7 times compared with those in 1992, respectively. Level-2 and Level-3 disappeared in 1999–2011 due to the increased green space and ventilation corridors in high-rise residential areas. Building density was rapidly lowered, and environment in residential areas was improved, so the incidence of respiratory diseases dropped (23). Level-4, which has the highest respiratory diseases mortality, occurred in 2018. Hence, UHI rapidly deteriorated in local regions, and the influences of UHI on health of respiratory system was continuously worsened.

**Table 2:** Changes in PLAND in 1992–2018

***Variabl***	***1992***	***1999***	***2001***	***2006***	***2009***	***2011***	***2013***	***2018***
Level-0	98.911	99.904	99.969	99.917	99.945	99.824	99.084	82.14
Level-1	0.7096	0.0963	0.0307	0.0829	0.0546	0.1757	0.7147	11.214
Level-2	0.3712						0.1962	5.4568
Level-3	0.0081						0.0056	1.1202
Level-4								0.0683

Table 3 shows the changes in PD in 1992–2018. In the study area, PD generally increased continuously, indicating the significantly intensified fragmentation and decentralized distribution of different levels. Level-1 presented an exponential growth and soared up after 2011. In 2013–2018, the fragmentation of the influenced patches, closely related to high-density urban development and green space construction, was continuously intensified. District construction in urban areas brought new concentrated regions of respiratory diseases. The cold source formed by the construction of green space relieved the effects of UHI on respiratory system.

**Table 3:** Changes in PD in 1992–2018

***Variable***	***1992***	***1999***	***2001***	***2006***	***2009***	***2011***	***2013***	***2018***
Level-0	0.0005	0.0005	0.0005	0.0005	0.0005	0.0005	0.0005	0.0019
Level-1	0.001	0.0005	0.0005	0.0019	0.0014	0.0019	0.0082	0.0236
Level-2	0.001						0.0024	0.0221
Level-3	0.0005						0.001	0.0106
Level-4								0.001

Table 4 shows the changes in COHESION in 1992–2018. On the whole, the connectivity of Level-0 slightly decreased and formed different levels of influenced areas. Level-1 gradually declined and then increased. The influences of UHI intensity on respiratory diseases at low levels were significantly correlated with the expansion of urban built-up area. Level-2 and Level-3 continued to increase in 2013–2018. The influenced areas at high level, which were against the fast heat dissipation in patches, were agglomerated. The mortality of respiratory diseases increased as a result of the gradual rise of air temperature. Level-4 began to form in 2018, but with the lowest connectivity. The spots of regions with high incidence of respiratory diseases caused by UHI intensity scattered in the area.

**Table 4:** Changes in COHESION in 1992–2018

***Variable***	***1992***	***1999***	***2001***	***2006***	***2009***	***2011***	***2013***	***2018***
Level-0	100	100	100	100	100	100	100	99.992
Level-1	99.246	97.945	96.31	95.906	95.457	96.908	97.76	99.631
Level-2	98.596						97.37	99.168
Level-3	92.768						90.336	98.104
Level-4								96.857

### Changes in landscape level of landscape pattern

**Fig. 3:**
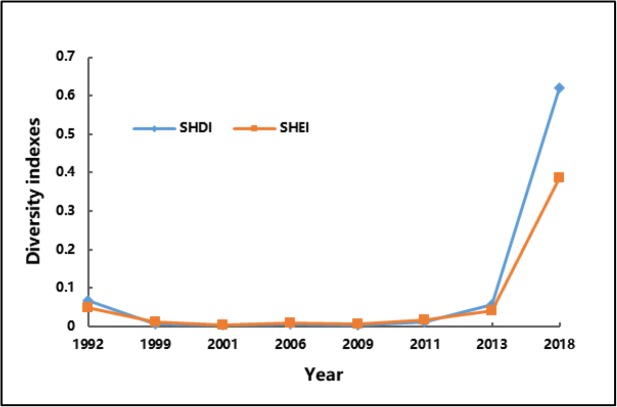
Variation curves of SHDI and SHEI in 1992–2018

Fig. 3 shows the changes in SHDI and SHEI in 1992–2019. SHDI and SHEI presented a falling tendency first, but then rose rapidly. They declined in 1992–1999 and slightly fluctuated in 1999–2011, but finally significantly increased in 2013–2018. Generally, SHDI and SHEI increased continuously. Hence, the complexity of the influence UHI intensity yielded on respiratory diseases increased, accompanied with enriching classes of patches. The area of the influenced patches significantly expanded, and a new high-intensity patch was formed due to the rapid urbanization in Tianjin. All levels of affected areas gradually expanded to the entire city.

**Fig. 4:**
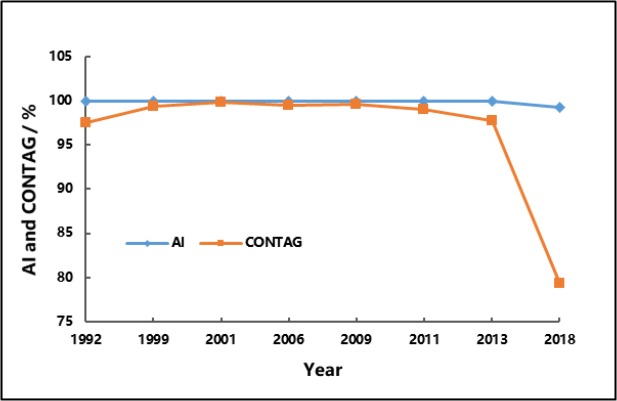
Variation curves of AI and CONTAG in 1992–2018

Fig. 4 shows the changes in AI and CONTAG in 1992–2018. AI generally but also slightly declined, indicating the relatively evident concentration of influenced areas. CONTAG significantly dropped in the late period, indicating that the high fragmentation of landscape and connectivity of influenced areas decreased. The influenced patches at different levels diffused from central areas to suburbs, demonstrating that residents in Tianjin were facing risks of respiratory diseases, and such risks decreased in 1992–2009 but significantly increased after 2013. The influenced areas were mainly distributed in Heping, Hebei, Hedong, Hexi, Nankai, and Hongqiao Districts.

### Response Mechanism of UHI to Respiratory System Health

Air temperature can indirectly induce respiratory diseases. When extreme weather happens, the self-regulatory mechanism of the human body cannot adapt to the external stimuli, and the activity of viruses is strengthened under extreme temperatures, thereby further increasing the incidence or mortality of respiratory diseases. Multiple research found that respiratory syncytial viruses were spawned at approximately 24 °C–30 °C and spread in a large scale after virus infection (8), and air temperature could influence inflammation pathways or pathophysiological reactions. For example, the vasoconstriction of respiratory mucosa and inhibition of immune response could influence respiratory tract infection (24).

Increase in air temperature significantly affects patients. Under high air temperature, patients with COPD suffer from hyperpnea and pulmonary hyperinflation, which can further cause dyspnea. This situation can inhibit anti-infection immunity functions, thereby exacerbating the symptoms of respiratory diseases (25–6). The continuous increase in air temperature can easily induce the inflammation response of the respiratory system. Increased inflammatory substances in bronchoalveolar lavage fluid, such as karyocytes, neutrophil granulocyte and chemotactic factor, damage air passage and make the respiratory tract infected by bacteria, fungi, and viruses. As a result, symptoms of asthma and COPD are intensified (27).

The high mortality of respiratory diseases in summer is attributed to the great temperature difference between indoor and outdoor environments. The upper respiratory tract of the human body is attacked by cold air produced by air conditioning when people go indoors. Such attack causes reflex spasm on the trachea and bronchia which originally have a high response state. Relatively closed environment and poor air ventilation in the air conditioning room affect the health of the respiratory system. Meanwhile, catching a cold after sweating increase the incidence of heat-type cold and viral influenza.

## Discussion

The study shows that UHI yields an impact on respiratory diseases in class level, which mainly can be found in the seriousness, size and fragmentation of affected areas. In landscape level, the impact is shown in the complexity of space and spatial embedding. Moreover, influences are concentrated in the central regions and scatter around in suburb regions. The conclusion proves that the influences of UHI on respiratory diseases demonstrate landscape–pattern–process characteristics, echoing with other relevant case studies done through landscape fragmentation and diversity indexes on the tropical disease Buruli ulcer (28). Therefore, landscape pattern index is applicable in studying the impact of the environment on diseases.

The study of the correlation between air temperature and mortality during warm seasons (April to September) in Porto and Lisbon found that the all-cause mortality in these two regions increases by 3% (95% CI:2.0%–3.9%) and 5.6% (95% CI:4.6%–6.6%) with every 1 °C rise of maximum daily temperature, respectively. Such correlation is the most significant in respiratory diseases (29). In this study, the influences of UHI on the mortality of respiratory diseases are intensified with the increase in UHI, concerning more urban citizens and built-up areas. Urbanization improved dramatically in Tianjin in 2013–2018. Air temperature continues to increase, and human body’s functions are seriously affected. The failure of the human body to adapt to high temperature after exposure induces respiratory diseases and increases additional mortality in urban areas.

Furthermore, in this study, the strong spatial heterogeneity of the degree of UHI influences on respiratory diseases is found. UHI intensity significantly affected respiratory diseases in 1992 at three levels. However, only a small Level-1 influenced area was observed in 1999–2011, but the level and size of the influenced areas significantly expanded in 2011–2018. The health of urban residents is significantly influenced as a response to the increasing UHI intensity with the continuous expansion of urban space. Therefore, the level and size of affected areas of respiratory diseases continue to expand. Although the health of residents is protected by the improving medical services, many studies highlight that high temperature in summer is still closely related to incidence and mortality of respiratory diseases (9). Therefore, optimizing landscape pattern in urban green space, reducing exposure to high temperature in summer, and further improving the health condition of residents can provide reference for healthy urban planning and construction.

In this study, the landscape pattern indexes of influences of UHI on the health of respiratory system are used as evaluation indexes, which can reflect the influences of temperature change on macroscopic landscape pattern. However, this study only analyzes the spatial distribution characteristics of influences of UHI on respiratory diseases in summer excluding other seasons.

## Conclusion

With the continuously intensifying UHI, the level and area of UHI influence on respiratory diseases generally increases. Relevant influence can be divided into two stages: long-term low-level reduction and short-term rapid increases.

On the class level, the connectivity of influence areas at low-level is relatively high, and the spatial distribution is relatively concentrated. However, the influence areas at high-level are distributed in groups. The features of overall landscape are fragmentation and discontinuity, and concentration in the central area in terms of space. UHI increases the daily average air temperature in summer, thereby influencing the conditions for survival and spread of bacteria and viruses, as well as the physiological reactions of patients. As a result, the mortality of respiratory diseases is worsened.

## Ethical considerations

Ethical issues (including plagiarism, Informed Consent, misconduct, data fabrication and/or falsification, double publication and/or submission, redundancy, etc.) have been completely observed by the authors.

## References

[1] Li Y Cheng Y Cui G (2014). Association between high temperature and mortality in metropolitan areas of four cities in various climatic zones in China: a time-series study. Environ Health, 13:65. 25103276 10.1186/1476-069X-13-65 PMC4237799

[2] Xie P Wang YL Peng J (2015). Health related urban heat wave vulnerability assessment: research progress and framework. Prog Geo, 34(2): 165–74.

[3] Qin DH (2014). Climate change science and sustainable development. Prog Geo, 33(07): 874–83. [In Chinese]

[4] Stafoggia M Forastiere F Agostini D (2008). Factors affecting in-hospital heat-related mortality: a multi-city case-crossover analysis. J Epidemiol Community Health, 62(3): 209–15. 18272735 10.1136/jech.2007.060715

[5] Zanobetti A O’Neill MS Gronlund CJ (2013). Susceptibility to mortality in weather extremes: effect modification by personal and small-area characteristics in a Multi-City Case-Only Analysis. Epidemiology, 24(6): 809–19. 24045717 10.1097/01.ede.0000434432.06765.91 PMC4304207

[6] Meng X Zhang YH Zhao ZH (2012). Temperature modifies the acute effect of particulate air pollution on mortality in eight Chinese cities. Sci Total Environ, 435-436:215–21. 22854092 10.1016/j.scitotenv.2012.07.008

[7] Michelozzi P Accetta G De Sario M (2009). High Temperature and Hospitalizations for Cardiovascular and Respiratory Causes in 12European Cities. Am J Respir Crit Care Med, 179(5):383–9. 19060232 10.1164/rccm.200802-217OC

[8] Mo YZ Zhen YA Tao H (2012). Relationship between daily mean temperature and emergency department visits for respiratory diseases: a time-series analysis. Beijing Da Xue Xue Bao Yi Xue Ban, 44(3):416–20. [In Chinese] 22692314

[9] Yue M Wang SG Xie JJ (2018). Study about the impact of environment conditions on respiratory diseases and prediction in Zuiyi City. China Environ Sci, 38(11):4334–47. [In Chinese]

[10] Liu YX Peng J Wang YL (2017). Relationship between urban heat island and landscape patterns: From city size and landscape composition to spatial configuration. Acta Ecol Sin, 37(23):7769–80. [In Chinese]

[11] Zhao QY Sun YL Wang ZL (2014). Analysis on changes of urban heat island landscape pattern in the urbanization process of Tianjin. J Tianjin Norm Univ (Nat Sci E), 34(02):49–55. [In Chinese]

[12] Tran PM Waller L (2013). Effects of Landscape Fragmentation and Climate on Lyme Disease Incidence in the Northeastern United States. Eco Health, 10(4):394–404. 24419663 10.1007/s10393-013-0890-y

[13] Liu H Weng Q (2009). An examination of the effect of landscape pattern, land surface temperature, and socioeconomic conditions on WNV dissemination in Chicago. Environ Monit Assess, 159(1–4):143–61. 19107566 10.1007/s10661-008-0618-6

[14] Li J Xu X Yang J (2017). Ambient high temperature and mortality in Jinan, China: A study of heat thresholds and vulnerable populations. Environ Res, 156(4):657–64. 28463825 10.1016/j.envres.2017.04.020

[15] Yue H Liu Y (2018). Comparison and analysis of land surface temperature retrieval algorithms based on Landsat 8 TIRS. Sci Technol Eng, 18(20):200–5. [In Chinese]

[16] Qin ZH Li WJ Zhang MH (2003). Estimating of the essential atmospheric parameters of mono-window algorithm for land surface temperature retrieval from Landsat TM6. Remote Sens Land & Resour, (02):37–43. [In Chinese]

[17] Zhang LB Cui QC Wang FX (2013). Study on Dynamic Changes of Vegetation Coverage of Yellow River Delta Based on NDVI. J Ludong Univ (Nat Sci E), 29(03):250-4+89. [In Chinese]

[18] Paynter S (2015). Humidity and respiratory virus transmission in tropical and temperate settings. Epidemiol Infect, 143(6):1110–8. 25307020 10.1017/S0950268814002702 PMC9507187

[19] Sheffield PE Landrigan PJ (2010). Global Climate Change and Children’s Health: Threats and Strategies for Prevention. Environ Health Perspect, 119(3):291–8. 20947468 10.1289/ehp.1002233 PMC3059989

[20] Chen ZG Zhu Y Wang Y (2014). Association of meteorological factors with childhood viral acute respiratory infections in subtropical China: an analysis over 11 years. Arch Virol, 159(4):631–9. 24114148 10.1007/s00705-013-1863-8

[21] Zhang Y Shang KZ Sun H (2018). Correlation analysis between the death toll from respiratory system and circulatory system diseases and meteorological factors in Nanjing City. J Lanzhou Univ (Nat Sci), 50(1):59–65. [In Chinese]

[22] Song ZQ Wang YL (2004). Progress in research on ecological impact of urban landscape structure. Prog Geo, 23(2):97–106. [In Chinese]

[23] Huang HC Yun YX Wang SZ (2014). An analysis of landscape evolution for the thermal comfort degree affected by urban heat island effect. J Harbin I Technol, 46(10):99–105. [In Chinese]

[24] Buckley JP Richardson DB (2012). Seasonal modification of the association between temperature and adult emergency department visits for asthma: a case-crossover study. Environ Health, 11(1):55. 22898319 10.1186/1476-069X-11-55 PMC3489538

[25] Zanobetti A Schwartz J (2009). The effect of fine and coarse particulate air pollution on mortality: a national analysis. Environ Health Perspect, 117(6):898–903. 19590680 10.1289/ehp.0800108 PMC2702403

[26] Delamater PL Finley AO Banerjee S (2012). An analysis of asthma hospitalizations, air pollution, and weather conditions in Los Angeles County, California. Sci Total Environ, 425(9):110–8. 22475217 10.1016/j.scitotenv.2012.02.015 PMC4451222

[27] Luo B Luo XF Shi HX (2014). Interactive effects of ambient temperature and ambient particulate matter on respiratory system: a review of recent studies. J Environ Health, 31(6):551–5. [In Chinese]

[28] Wu JY Smithwick EAH (2016). Landscape fragmentation as a risk factor for Buruli ulcer disease in Ghana. Am J Trop Med Hyg, 95(1):63–9. 27185767 10.4269/ajtmh.15-0647 PMC4944711

[29] Almeida S Casimiro E Analitis A (2012). Short-term effects of summer temperatures on mortality in Portugal: a time-series analysis. J Toxicol Environ Heal A, 76(7):422–8. 10.1080/15287394.2013.771550 23738393

